# Complete chloroplast genome of *Micractinium singularis* MM0003 (Chlorellaceae, Trebouxiophyceae)

**DOI:** 10.1080/23802359.2020.1715897

**Published:** 2020-01-24

**Authors:** Seung-Woo Jo, Kyeong Mi Kim, Nam Seon Kang, Jung A Lee, Eun Song Kim, Moongeun Yoon, Ji Won Hong, Ho-Sung Yoon

**Affiliations:** a Department of Energy Science, Kyungpook National University, Daegu, Korea;; b School of Life Sciences, Kyungpook National University, Daegu, Korea;; c Department of Taxonomy and Systematics, National Marine Biodiversity Institute of Korea, Seocheon, Korea

**Keywords:** Complete chloroplast genome, *Micractinium singularis*, Chlorellaceae, Trebouxiophyceae

## Abstract

The chloroplast genome of *Micractinium singularis* MM0003 was completely sequenced. This plastome has 139,597 bp in length and consists of 106 genes including 77 protein-coding, 3 rRNA, and 26 tRNA genes. The overall GC content of the genome is 34.0%.

*Micractinium* Fresenius is a globally distributed genus inhabiting a variety of aquatic systems. Chae et al. (2019) recently described a new species, *M*. *singularis* H. Chae, H-G. Choi & J. H. Kim (Chlorellaceae, Trebouxiophyceae), isolated from Deception Island, South Shetland Islands, Antarctica. The holotype of this species is *M*. *singularis* KSF0094. It is assumed that *M*. *singularis* and its close relatives possess cold-tolerant properties. In this study, the complete plastome of *M*. *singularis* MM0003 was determined for the first time.

*Micractinium singularis* MM0003 was isolated from Janghang Harbor, Seocheon, Korea (36°00′23.96″N 126°41′23.52″E). Morphological features and molecular phylogenetic evidences inferred from the small subunit 18S rRNA and internal transcribed spacer sequences indicated that strain MM0003 belonged to this newly described species. *Micractinium singularis* MM0003 was deposited at the National Marine Biodiversity Institute of Korea (MABIK) and Korean Collection for Type Cultures (KCTC) under the accession numbers of MABIK-LP-00000134 and KCTC 13290BP, respectively. The culture was grown in BG-11 medium (UTEX, Austin, TX, USA) at 18 °C under cool fluorescent light (approximately 40µmolem^−2^s^−1^) in a light:dark cycle (14:10 hrs) for 4 weeks until growth was apparent. Biomass was harvested by centrifugation at centrifugation at 2063 ×*g* (1580 R; Labogene, Daejeon, Korea). Whole genomic DNA was extracted from the sample using a DNeasy Plant Mini Kit (Qiagen, Hilden, Germany) followed by the preparation of a library using an MGIEasy DNA Library Prep Kit V1 (BGI, Shenzhen, China) according to the manufacturer’s instruction. Whole genome sequencing was performed using BGISEQ-500 (BGI, China) sequencer and raw data were filtered to obtain >10 Gb clean data per each sample. *De novo* plastome assembly was carried out using NOVOPlasty v3.6 software (Dierckxsens et al. 2017). The size of the circular plastome produced is 139,597 bp (GenBank accession number MN894287) which is larger than that of the previously reported *M*. *pusillum* (115,638 bp, GenBank accession number MN649872) and slightly smaller than that of *M*. *conductrix* (149,364 bp, GenBank accession number KY629619). The nucleotide composition is 33.2% A, 32.8% T, 16.5% G, and 17.5% C. The overall GC content is 34.0%. The *M*. *singularis* plastome contains 106 genes, including 77 predicted protein-coding, 3 rRNA, and 26 tRNA genes. All 77 genes were revealed as complete protein-coding genes, which all of them were started with ATG as a start codon (except psbK, which starts with GTG) and ended by a stop codon (73 genes by TAA, 4 genes by TAG). It was found that there was only 1 case of gene-overlapping with a size of 52 bp and 26 tRNA genes ranged from 66 to 87 bp in length. Phylogenetic analysis was performed by using PhyML 3.0 with 14 reported chloroplast sequences (Fan et al. 2017) belonging to the Trebouxiophyceae family and the phylogenetic tree was visualized by FigTree v1.4.4. The result indicated that its phylogenetic position is within Trebouxiophyceae (Figure 1). This new sequence information would contribute to a better understanding of the phylogenetic relationships of the *Micractinium* species and plastome genome diversity and evolution in the family Trebouxiophyceae.

**Figure 1.**
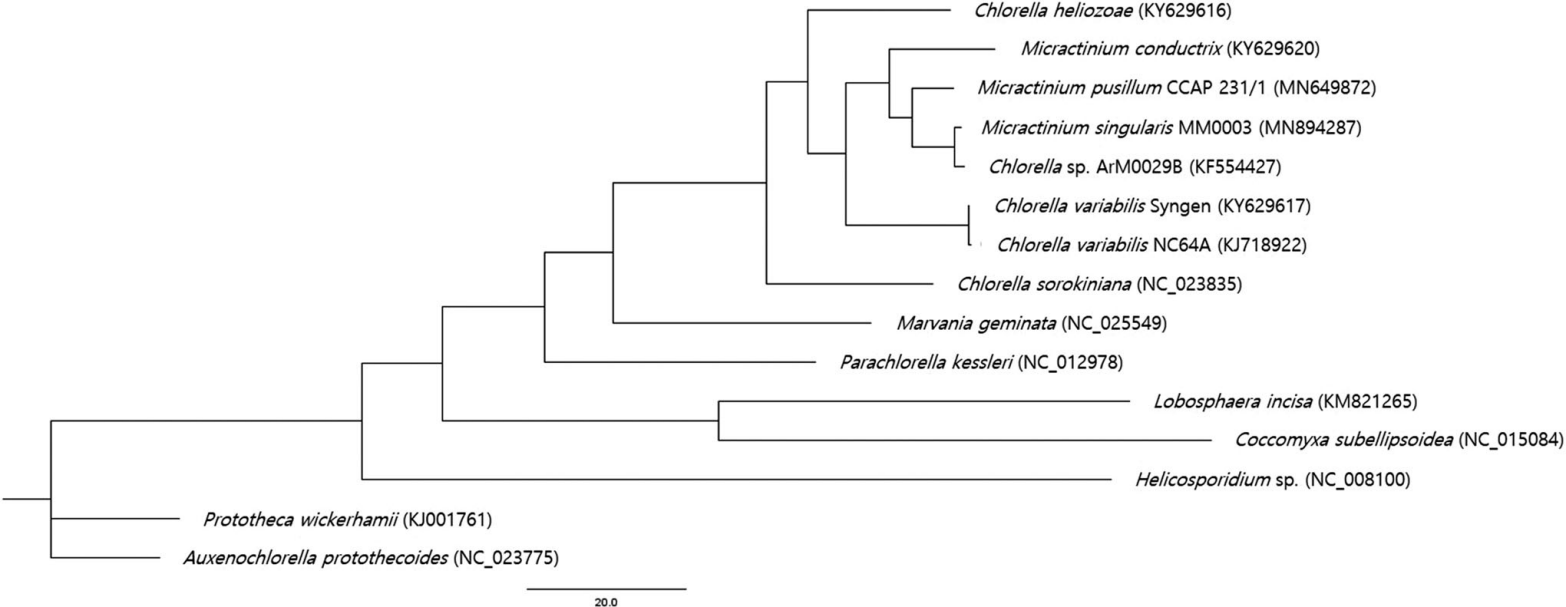
Maximum-likelihood phylogenetic tree of *M. singularis* MM0003 and 14 other species. GenBank accession numbers were indicated in the parentheses.
